# Prediction of Disease Progression to Upfront Pembrolizumab Monotherapy in Advanced Non-Small-Cell Lung Cancer with High PD-L1 Expression Using Baseline CT Disease Quantification and Smoking Pack Years

**DOI:** 10.3390/curroncol30060419

**Published:** 2023-06-08

**Authors:** Ali Silver, Cheryl Ho, Qian Ye, Jianjun Zhang, Ian Janzen, Jessica Li, Montgomery Martin, Lang Wu, Ying Wang, Stephen Lam, Calum MacAulay, Barbara Melosky, Ren Yuan

**Affiliations:** 1 Department of Radiology, Faculty of Medicine, University of British Columbia, 2329 West Mall, Vancouver, BC V6T 1Z4, Canada; 2 BC Cancer, Vancouver Center, 600 West 10th Avenue, Vancouver, BC V5Z 4E6, Canada; 3 Department of Medical Oncology, Faculty of Medicine, University of British Columbia, 2329 West Mall, Vancouver, BC V6T 1Z4, Canada; 4 Department of Statistics, Faculty of Science, University of British Columbia, 2329 West Mall, Vancouver, BC V6T 1Z4, Canada; 5 Department of Thoracic/Head and Neck Medical Oncology, MD Anderson Cancer Center, The University of Texas, Houston, TX 77030, USA; 6 Integrative Oncology, BC Cancer Research Centre, 675 West 10th Avenue Vancouver, BC V5Z 1L3, Canada; 7 Department of Respirology, Faculty of Medicine, University of British Columbia, 2329 West Mall, Vancouver, BC V6T 1Z4, Canada

**Keywords:** non-small-cell lung cancer, baseline CT, smoking pack years, pembrolizumab, tumor response, survival, PDL1

## Abstract

Health Canada approved pembrolizumab in the first-line setting for advanced non-small-cell lung cancer with PD-L1 ≥ 50% and no EGFR/ALK aberration. The keynote 024 trial showed 55% of such patients progress with pembrolizumab monotherapy. We propose that the combination of baseline CT and clinical factors can help identify those patients who may progress. In 138 eligible patients from our institution, we retrospectively collected their baseline variables, including baseline CT findings (primary lung tumor size and metastatic site), smoking pack years, performance status, tumor pathology, and demographics. The treatment response was assessed via RECIST 1.1 using the baseline and first follow-up CT. Associations between the baseline variables and progressive disease (PD) were tested by logistic regression analyses. The results showed 46/138 patients had PD. The baseline CT “number of involved organs” by metastasis and smoking pack years were independently associated with PD (*p* < 0.05), and the ROC analysis showed a good performance of the model that integrated these variables in predicting PD (AUC: 0.79). This pilot study suggests that the combination of baseline CT disease and smoking PY can identify who may progress on pembrolizumab monotherapy and can potentially facilitate decision-making for the optimal first-line treatment in the high PD-L1 cohort.

## 1. Introduction

Lung cancer remains the leading cancer-related death globally, with the 5-year survival at 22% [1,2]. Most patients are diagnosed with advanced non-small-cell lung cancer (NSCLC) [1], for which the current first-line treatment decisions are based on the presence of genetic aberrations, including the epidermal growth factor receptor (EGFR), anaplastic lymphoma kinase (ALK), UR2 sarcoma virus oncogene homolog 1 (ROS1), B-Raf proto-oncogene serine/threonine kinase (BRAF), Kirsten rat sarcoma virus (KRAS), and neurotrophic tropomyosin-related kinases (NTRK), amongst other targetable mutations [3]. Immune checkpoint inhibitors (ICIs) have fulfilled their promise for advanced-stage lung cancer therapy in patients without a targetable mutation. Clinical trials have shown that ICIs, either as a single agent (e.g., pembrolizumab) [4,5] or a combination of immunotherapy and chemotherapy [6,7,8,9], are superior to platinum chemotherapy alone in first-line setting.

The KN024 trial demonstrated that pembrolizumab monotherapy improved the overall response rate (ORR) and overall and progression-free survival (OS and PFS) compared to platinum doublet chemotherapy (PDC) in the first-line setting for advanced NSCLC patients with a high PDL1 expression (i.e., ≥50%) and no EGFR/ALK aberration [5]. Health Canada approved pembrolizumab in the first-line setting for this patient population in July 2017 [3]. The response rate to the single agent pembrolizumab in KN024 was 45%, and according to the updated KN024′s 5-year survival data, 32% of patients were alive at five years, and the long-term survival was mainly driven by the responders [10]. However, the remaining 55% of patients did not respond to pembrolizumab monotherapy and therefore might need alternative therapy, such as combination chemotherapy and ICI. Single pembrolizumab, despite being the current standard first-line choice, may potentially jeopardize the optimal first-line treatment regimen in a selected subpopulation of high PDL1 patients. The clinical dilemma of choosing the single agent pembrolizumab or a triplet chemotherapy and ICI in advanced NSCLC with a high PD-L1 expression could be avoided if we could distinguish the two subsets before treatment initiation.

The goal of this study was to identify, in advanced NSCLC patients with PD-L1 ≥ 50% and no EGFR/ALK aberration, which baseline features could predict a patient’s future disease progression assessed radiographically at the first follow-up CT. There have been no clinical radiologic studies that have specifically correlated the baseline CT disease features to the treatment response. We propose that the combination of a patient’s baseline CT findings and clinical factors, including smoking pack years, can predict the treatment response and can be used to guide first-line treatment decisions in this PD-L1 high cohort.

## 2. Materials and Methods

### 2.1. Patients

We obtained approval from the institutional review board for this study. The review board waived the requirement for written informed consent for this study, as it is a retrospective study with minimal risk. Patients were identified using a retrospective data access request for NSCLC patients referred to the single center at British Columbia Cancer from 1 January 2015, to 31 May 2019. We consecutively recruited patients meeting the following eligibility criteria: males and females from all races and ethnic groups who received first-line monotherapy with pembrolizumab for stage IIIB or IV NSCLC without EGFR mutation or translocation of the ALK gene and with PD-L1 expression ≥50% (as determined by the Stand-of-Care IHC assay used at our institution). All had adequate hepatic and renal function. All must have had a baseline staging CT ≤ 6 weeks prior to starting treatment and the first follow-up (FU) CT within 9 to 12 weeks after treatment initiation for the response assessment. Patients were excluded if they received concurrent chemotherapy, any other form of immunotherapy, or radiation; had an active, known, or suspected autoimmune disease; or received long-term immunosuppressive therapy or systemic corticosteroids (requiring more than 10 mg prednisone/day or equivalent).

The baseline demographic characteristics were collected, including age, sex, Eastern Cooperative Oncology Group performance status (ECOG), and smoking status and pack years (PY), where a light to never smoker was defined as a patient who had smoked fewer than 100 cigarettes. Tumor histologic subtype and cancer stage at presentation were also collected.

### 2.2. CT Scan Acquisition and Review

The baseline and first FU CT scans were acquired according to the standard body scanning protocols at our institution. All CT scans used a GE Light Speed CT scanner (GE Healthcare, Milwaukee, WI, USA), with an intravenous iodinated contrast injection obtained at the portal venous phase for the chest, abdomen, and pelvis, with the images reconstructed using a 2 or 2.5 mm slice thickness. Imaging of the brain included pre- and post-IV contrast scans from a CT and/or MRI. All follow-up scans were acquired using the same protocol.

Two board-certified oncology radiologists, with 8 and 20 years of experience in tumor burden measurements/response assessments, performed the imaging analysis while blinded to the patient outcome data. Any question from the imaging analysis was resolved by the consensus of the two radiologists. For the baseline CT, the radiologists measured the largest dimension of the primary lung tumor, documented the sites of metastatic disease, and summed the total “number of involved organs” as an estimate of the disease burden (Table 1). When the findings from the CT were not specific for malignance/metastasis, additional information, including other imaging modalities (e.g., PETCT, MRI, and US) or tissue diagnosis results from biopsies, was used.

Next, the baseline and first FU CT were compared to assess the treatment response as per the Response Evaluation Criteria in Solid Tumors criteria (RECIST v1.1) [11], which included measuring up to five target tumor lesions (maximum two lesions per organ) with the longest diameters. Pathologically enlarged lymph nodes were considered target lesions if they measured ≥15 mm on the short axis.

According to RECIST V1.1, progressive disease (PD) was at least a 20% increase in the sum of the diameters of the target lesions (with the sum also demonstrating an absolute 5 mm increase in size). A complete response (CR) was the disappearance of all target lesions and reduction of pathological lymph nodes to a <10 mm short axis. A partial response (PR) was at least a 30% decrease in the sum of the diameters of the target lesions. A stable disease (SD) had neither sufficient shrinkage to qualify for PR nor was sufficient to qualify for PD. Patients were dichotomized into the “progressive disease, PD” group or “disease control, DC” group if they demonstrated CR, PR, or SD.

### 2.3. Statistical Analysis

The primary endpoint for this study was progressive disease (PD). The explanatory variables were baseline characteristics, including the primary lung tumor size and “number of involved organs”, from the CT and patient’s age, sex, smoking status and pack years, and ECOG.

To illustrate the relationship between PD and the baseline characteristics, we first compared the baseline characteristics between two response groups (PD vs. DC) using the Kruskal–Wallis test for continuous, and chi-square or Fisher’s exact test for categorical, variables.

Then, the associations between the baseline characteristics and PD were examined using uni- and multivariate (MV) logistic regression analyses. To restrict the number of variables in the MV analysis, we considered only the variables that showed a trend of significant associationd with PD (*p* ≤ 0.2) in the univariate analysis. The combination of variables in the final best fit MV model was determined by using the area under the curve (AUC) criterion, such that the selected combination achieved the best AUC index across the different combinations. For the final MV logistic model with the best AUC, its performance in classifying PD vs. DC was assessed using the receiver operating characteristic (ROC) curve, with which the sensitivity (SN) and specificity (SP) at the optimal cut-off were reported. Two commonly used cut-offs were used [12] (i) the “Youden” cut-off point that maximizes the distance to the identity (diagonal) line of the ROC plot, which simultaneously maximizes the sum of the SN and SP [13], and (ii) the “topleft” cut-off, the point closest to the top-left part of the ROC curve with the best and balancing SN or SP.

Then, individual patient’s “risk of progression” was calculated using the best MV logistic model (patients were excluded from the MV model if there were missing data in any of the explanatory variables). Patients were separated into the “low-” or “high-risk of progression” group by comparing their calculated risk to the Youden or topleft cut-off (i.e., high- vs. low-risk was defined if the calculated risk was higher or lower than the Youden or topleft cut-off).

The progression-free survival (PFS) and overall survival (OS) between the predicted high- vs. low-risk progression groups were compared by Kaplan–Meier plots using log-rank tests. PFS was defined as the time from starting pembrolizumab to the earliest CT date that showed disease progression or the latest available CT date for patients with no sign of progression. OS was defined as the time from starting pembrolizumab to death or the date of the last follow-up.

All statistical analyses were performed with R software (Version 4.1.3. 2022-03-10. R: The R Project for Statistical Computing (r-project.org). All statistical tests were two-sided and considered significant with a *p*-value < 0.05.

## 3. Results

### 3.1. Summary of Patient’s Baseline Characteristics and Treatment Response at the 1st Follow-Up CT

A total of 138 eligible patients (53% female) were included, with a median age of 73 years ranging from 61 to 91 years. ECOG ranged from 0 to 4, with 81/138 (59%) having an ECOG ≤ 1 and 53/138 (38%) with ECOG ≥ 2, while 4/138 (3%) had an unknown ECOG status. Most patient (92%) were current or ex-smokers (*n* = 31 and 96, respectively), 7/138 (5%) were never smokers, and 4/138 (3%) had an unknown smoking status. Pack years (PY) data were available for 122/127 smokers, and nearly half of the patients (57/122, 47%) had >40 PY (median pack years: 40; Q1–Q3: 28–50). Non-squamous was the main histologic subgroup (116/138, 84%), and the remaining were squamous (19/138, 14%) or not otherwise specified (NOS) (3, 2%).

At first FU CT, 46/138 (33%) showed PD and 92/138 (67%) patients had DC, including 1 (1%) CR, 51 PR (37%), and 40 SD (29%). The objective response rate (ORR) was 38% (ORR = CR + PR/138). The baseline variables are summarized and compared between the PD and DC groups in Table 1.

### 3.2. Comparison of the Baseline Variables between Patients with PD vs. DC

Descriptive analyses of 138 patients are shown in Table 1. The PD group was significantly enriched with patients with ≤40 smoking PK compared to the DC group (31/46, 67% vs. 41/92, 44%, *p* = 0.0031). Patients in the PD group had a poorer performance compared to the DC group (ECOG ≥ 2: 52% in PD vs. 32% in DC, *p* = 0.0153). Patients who demonstrated PD had a greater “number of involved organs” with metastasis on the baseline CT (a median of three vs. two organs per patient in the PD vs. DC group, *p* = 0.0015). More patients in the PD group had liver (22% in PD vs. 7% in DC, *p* = 0.0119) or bone metastasis (57% in PD vs. 24% in DC, *p* = 0.0003). Between PD and DC, no significant difference was found in age (*p* = 0.2129), sex (*p* = 0.2785), tumor histology (*p* = 0.1923), primary lung tumor size, i.e., T stage (*p* = 0.4821), or metastatic involvement in organs other than the liver and bone (all *p* > 0.05).

### 3.3. Association between Baseline Variables and Progressive Disease (PD)

Table 2 shows unadjusted and adjusted logistical regression testing the association between the baseline characteristics and PD. The final (i.e., adjusted) multivariate (MV) logistic model included the following baseline variables: age, sex, smoking pack years, ECOG and “number of involved organs” on the baseline CT. the data suggested that, after adjusting for other predictive factors, patients with no smoking history and a higher “number of involved organs” at the baseline CT more likely developed PD when receiving pembrolizumab. Compared with non-smokers, the odds of PD were 88% lower in smokers with ≤40 PY and 96% lower in those with >40 PY, indicated by the adjusted odds ratios of 0.12 and 0.04, respectively, in Table 2 (both *p* < 0.05). These data suggested smoking was associated with the response to pembrolizumab, and there was a probable dose–response effect between pack years and the response to pembrolizumab such that, compared to those with lighter pack years (i.e., ≤40), those with >40 pack years were at a lower risk to progress. Although having a poorer performance status (i.e., ECOG ≥2) was associated with PD in the univariate analysis, this association was not statistically significant in the MV model after adjusting for other predictors (*p* = 0.1357).

### 3.4. Performance of the MV Logistic Regression Model in Classifying PD or DC

**Figure 1:**
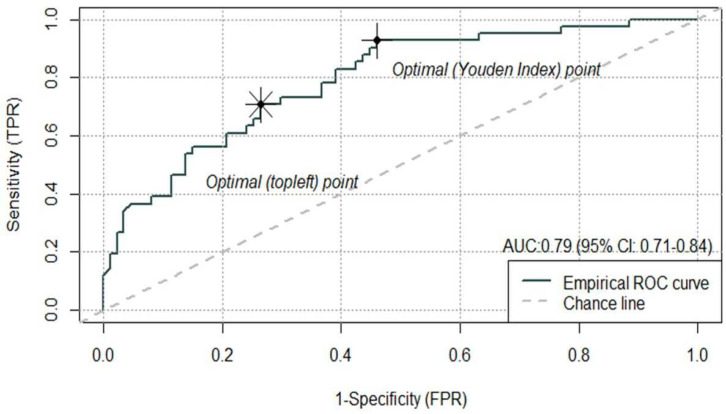
Performance of the MV logistical regression model (AUC at 0.79, 95% CI: 0.71–0.87). The Youden cut-off on the ROC curve was 0.20, and the “topleft” cut-off on the ROC was 0.33.

Ten patients (7% of 138) were excluded in this step due to missing data in either smoking or ECOG, and hence, the final model was conducted on 128 patients. The final MV logistic model demonstrated a good classification performance with an AUC of the ROC at 0.79 (95% CI: 0.71–0.84) (Figure 1). The Youden cut-off for the ROC curve was 0.20, with the corresponding SN and SP being 0.93 and 0.54 while the “topleft” cut-off was 0.33, which yielded a balance between the SN and SP (0.71 and 0.74, respectively).

### 3.5. Individual Predicted “Risk of Progression” and Predicted “High- vs. Low-Risk of Progression” Groups

An equation to calculate a patient’s predicted “risk of progression” derived from the above MV model was as follows:

(1) $$\begin{array}{cc} \mathrm{Predicted}\;\mathrm{risk}\;\mathrm{of} & \mathrm{PD}=[1+\exp(-3.91-0.65\times I\left(\mathrm{Male}\right)\\ & +2.11\times I\left(\mathrm{Smokers}\leq 40\;\mathrm{packyears}\right)\\ & +3.17\times I\left(\mathrm{Smokers}>40\;\mathrm{packyears}\right)-0.67\times I\left(\mathrm{ECOG}\geq 2\right)\\ & +0.059\times \mathrm{Age}\;\mathrm{in}\;\mathrm{years}\\ & {-0.46\times \mathrm{No}.\;\mathrm{of}\;\mathrm{involved}\;\mathrm{organs}\;\mathrm{at}\;\mathrm{baseline}\;\mathrm{CT}]}^{-1}, \end{array}$$

where *I*(∙) is an indication function, which takes the value of 1 if the condition inside the parentheses holds and takes 0 if otherwise. For example, *I*(“Make”) = 1 if it is a make patient and *I*(“Make”) = 0 if female

Then, the patients were classified into the “low-” or “high-risk of progression” group if the calculated risk value was lower or higher than the Youden or topleft cut-off. When using the Youden cut-off, 93% (38/41) of PD patients were accurately captured in the “high-risk of progression” group, indicating a high sensitivity of the Youden index (0.93), compared to 71% (29/41) when using the “topleft” cut-off. In contrast, using the topleft cut-off, 74% (64/87) of patients who achieved DC were accurately captured in the “low-risk of progression” group, indicating a high specificity (0.74) of the topleft cut-off, compared to 54% (47/87) when using the “Youden” cut-off (Table 3).

**Figure 2:**
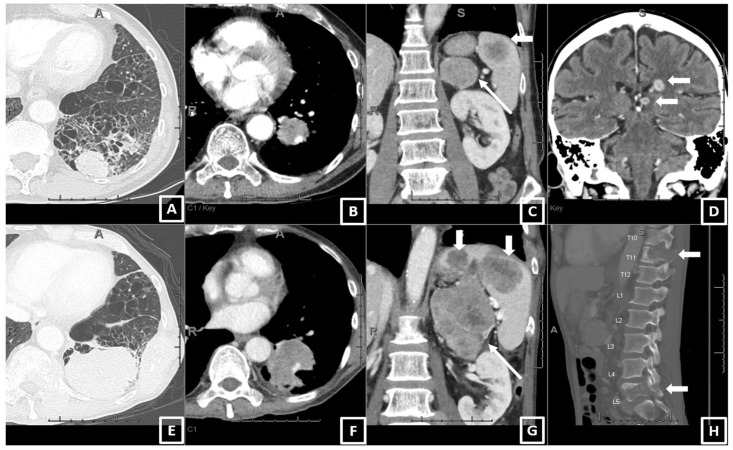
An example of a patient who had PD with pembrolizumab monotherapy and had a high value for the predicted risk of progression (0.93) using the prediction equation and the Youden cut-off. A 64-year-old male, current smoker, with 30 pack years, ECOG 2, diagnosed as stage IV NSCLC (adeno.). The baseline CT (**A**–**D**) showed (**A**) a 3.5 cm left lower lobe (LLL) primary lung mass, (**B**) a 2.2 cm LLL lobar adenopathy (also at the hilum and mediastinum, now shown), metastasis in the left adrenal and spleen (long and short arrows in (**C**)), and brain (**D**). After 3 months on pembrolizumab, the patient developed progressive disease on the 1st FU CT (**E**–**H**), including enlarging the LLL mass (6.1 cm, (**E**)), lobar adenopathy (3.2 cm, (**F**)), enlarging and new metastasis in the left adrenal and spleen (**G**), and worsening destructive bone metastases (**H**). The brain metastasis was radiated and improved. The patient died 7 weeks after the FU CT.

**Figure 3:**
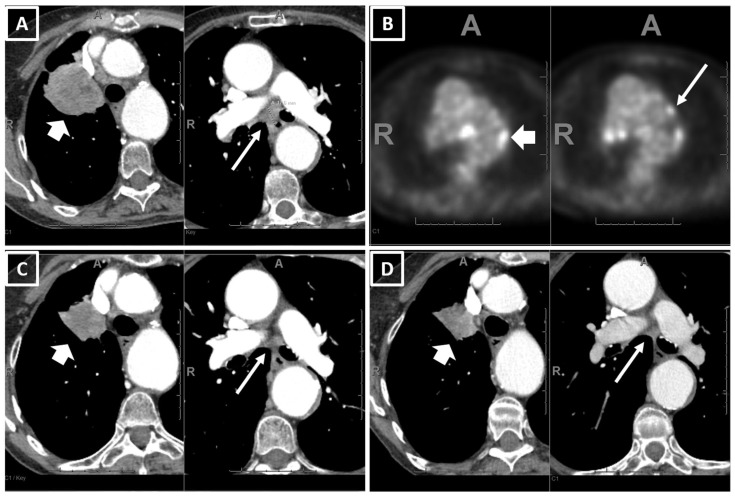
An example of a patient who had PR with pembrolizumab monotherapy and had a low value for the predicted risk of progression (0.03) using the prediction equation and the Youden cut-off. A 79-year-old female, ex-smoker, with 45 pack years, ECOG 1, diagnosed as stage IIIC NSCLC (adeno.). (**A**) The baseline CT showed a 6.0 (T3) right upper lobe primary lung tumor and 1.8 cm short axis subcarinal adenopathy (T3N2, short and long arrows in (**A**)), while (**B**) PETCT showed contralateral hilar and mediastinal adenopathy suggestive of N3 (short and long arrows in (**B**)). (**C**) The 1st FU CT after 11.5 weeks on pembrolizumab showed a partial response (PR) (RUL tumor decreased to 3.2 cm and LN decreased to a 0.7 cm short axis), and (**D**) the 2nd FU CT after 28 weeks showed a stable disease. The patient showed early signs of disease progression on the CT after 54 weeks on pembrolizumab.

Two example cases showing consistency between the real-world responses and predicted risk of progression are shown in Figure 2 and Figure 3.

### 3.6. Survival (PFS and OS) of the Predicted “High- vs. Low-Risk of Progression” Groups

**Figure 4:**
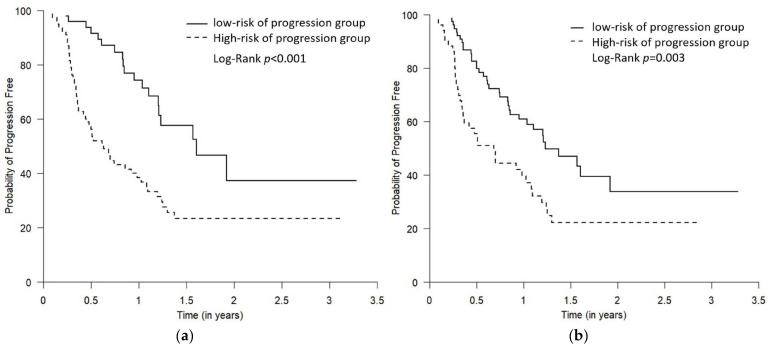
The Kaplan–Meier plots comparing PFS for the predicted low- and high-risk of progression groups. The two predicted risk of progression groups were defined using the Youden cut-off (**a**) and the “topleft” cut-off (**b**). The high-risk of progression group had statistically worse PFS using both Youden and “topleft” cut-offs, while the separation of the curves between the two groups was more drastic when the groups were defined using the Youden cut-off compared to using the “topleft” cut-off (*p* = 0.01 and 0.03, respectively).

**Figure 5:**
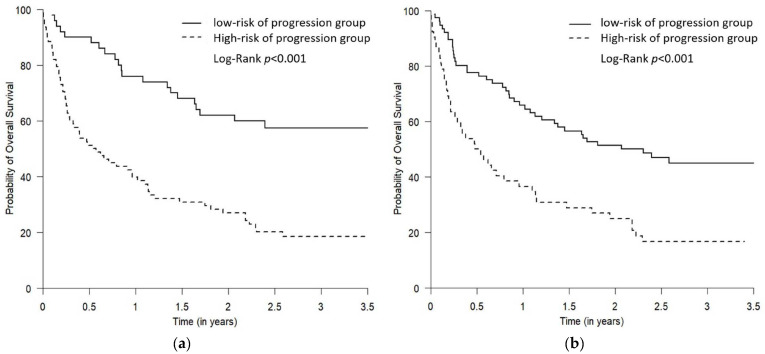
The Kaplan–Meier plots comparing OS for the predicted low- and high-risk of progression groups. The two predicted risk of progression groups were defined using the Youden cut-off (**a**) and the “topleft” cut-off (**b**). The high-risk of progression group had statistically worse OS using both Youden and “topleft” cut-offs, while the separation of the curves between the two groups was more drastic when the groups were defined using the Youden cut-off compared to using the “topleft” cut-off (both *p* < 0.001).

For PFS, the median follow-up time was 9 months (Q1–Q3: 4.3–14.8 months), and 56 were progression-free survivors. For OS, the median follow-up time was 19.2 months (Q1–Q3: 8.6–35.3 months), where 44 were survivors. The PFS and OS of the predicted “high- vs. low-risk of progression” groups were demonstrated by Kaplan–Meier plots (Figure 4 and Figure 5). The log-rank test showed that, compared to the predicted low-risk of progression group, the predicted high-risk of progression group had significantly worse PFS and OS using both Youden and “topleft” cut-offs, while the separation of the survival curves between the two groups was more drastic when the groups were defined using the Youden (Figure 4a and Figure 5a) compared to using the “topleft” cut-off (Figure 4b and Figure 5b).

## 4. Discussion

The present results show, in advanced NSCLC patients who are eligible for the currently standard first-line pembrolizumab monotherapy, their baseline burden of disease estimated with CT using the “number of involved organs” and smoking history/pack years as independent predictors of an adverse response (i.e., progressive disease, PD) to this treatment. Those who have no smoking history and a higher burden of disease on the baseline CT are at an increased risk of PD. Although smoking is associated with the response to pembrolizumab, compared to smokers with >40 pack years, those with ≤40 pack years have a higher risk for developing PD. For these high risk of progression patients, physicians may consider offering chemotherapy immunotherapy, or immunotherapy combinations upfront to provide more effective early control of the disease.

Studies have shown that some clinical parameters could facilitate response identification, such as age, gender, ECOG, and smoking status [5,7]. In this study, we used a multivariate logistic model to provide a comprehensive and personalized tool to estimate each patient’s risk of disease progression for upfront pembrolizumab monotherapy. Instead of using a single fixed cut-off value for each predictive variable across different individuals, this model incorporates multiple variables that are shown to significantly associate with progression, including those known ones (i.e., smoking status, ECOG, age, and gender) and other factors identified in our data (i.e., baseline CT “number. of involved organs” and smoking pack years). The estimated “risk of progression” by the model before commencing treatment can indeed capture patients into two distinct subsets with different survival outcomes.

From a clinical perspective, misclassifying patients belonging to the PD group (i.e., misclassifying a PD patient into the low-risk of progression group) is arguably more detrimental than misclassifying a DC patient. Therefore, we think the Youden cut-off may be preferred when using this MV prediction model to identify patients at a higher risk to progress, because the corresponding SN with the Youden cut-off is 0.93, which indicates that 93% of patients who are indeed in the PD group will be correctly predicted by the model as the “high-risk of progression group”, in contrast to only 71% if using the “topleft” cut-off.

In the current study, we used the “number of involved organs” (by diseases) counted using the baseline CT as the surrogate for the “overall disease burden”, and its positive association with PD suggested a relationship between widespread disease (i.e., more organ involvement) and a poor response to pembrolizumab monotherapy. This measure is not precisely how RECIST 1.1 describes the “disease burden”, as it does not consider the size of each target lesion. However, the multivariate logistic regression showed that it was significantly associated with the response outcome, while the “size of the primary lung tumor” was not. In addition, although it may not be as accurate as RECIST that was commonly used in the trial, in clinical practice, the “number of involved organs” could easily be quantified during the radiological review without labor-intensive measurements for each lesion. We think “the number of involved organs” on a CT could be a simple, meaningful, and practical measures that have the potential to guide the selection of therapy in clinical practice, with the consideration of adding chemotherapy or another immunotherapy. To our knowledge, this has not been reported, and this CT measure may expand our knowledge of the clinicoradiological characteristics of the subsets of this high PD-L1 cohort.

The impact of smoking history on the therapeutic response to immunotherapy has been reported, while there is limited data about the effect of pack years and the response in smokers. A recent meta-analysis supported that smoking history could be a simple index to guide the selection of potential responders to immunotherapy among NSCLC patients [14]. Yang et al. looked at the predictive factors in lung cancer patients who received neoadjuvant immunotherapy and found that the smoking signature is superior to PD-L1 in predicting a pathological response to immunotherapy. The authors showed that a heavy smoking history, defined as >40 pack years, was an independent predictor of a pathological response compared to a no or light smoking history [15]. This finding is in line with ours, which may suggest a dose–response effect between smoking pack years and the response to pembrolizumab.

Our study endpoint was the treatment response (i.e., PD vs. DC) rather than survival. We chose so, because treatment switching often relies on the response assessed radiographically at the FU CT in clinical practice, and it was the PD patients that we aimed to identify at the baseline before commencing treatment. In addition, we used the disease control (DC) rate (sum of CR, PR, or SD) to reflect the “response to treatment” [16], while it was the objective response rate (ORR, the presence of at least one confirmed CR or confirmed PR) that was used in prior clinical trials. We think the DC rate more closely reflects real-world situations, as, in clinical practice treatments, it will continue if the patient’s disease is controlled, including a stable disease (SD). The ORR to pembrolizumab monotherapy in our cohort was 38% (52 CR + PR, for a total 138 patients), lower than 45% in KN024, which might be due to more patients with a high disease burden and poor performance status in our cohort (i.e., 38% of patients had ECOG > 1, whereas all patients in KN024 had ECOG ≤1, and 35% had ECOG of 0).

Since the discovery of a checkpoint blockade through the anti-PD-1- and anti-PD-L1-based immunotherapies and their successful application in cancers, including lung cancer, identifying which patients are most likely to respond remains a challenge [17]. Despite being the most widely applied biomarker, growing evidence indicates that the expression level of PD-1/PD-L1 alone is insufficient to determine which patients should be offered ICIs [18,19,20]. Many efforts have been made to identify predictive biomarkers [17,21,22,23,24,25,26,27,28,29], such as using an invasive biopsy or liquid biopsy combined with time-consuming and labor-intensive laboratory and clinical testing, or the development of complicated predictive algorithms integrating genomic and tumor microenvironment features [30,31]. The present study provides a different approach using a patient’s clinical characteristics and non-complicated baseline CT data to identify the subgroup at risk of developing the progressive disease. Importantly, this risk prediction model can be made at the time of the baseline CT scan before commencing treatment and, hence, can help decision-making for the optimal first-line treatment in this high PD-L1 cohort.

From a medical imaging perspective, artificial intelligence (AI) and the CT imaging radiomic analysis have demonstrated remarkable progress and are expected to identify wide applications in assessing treatment responses. Future studies using radiomics in this cohort may identify radiomic features that can provide added value to the current results and serve as a “digital biomarker” to predict the responses to immunotherapy in this cohort.

The strength of our study is in having a homogeneous patient population (i.e., all patients had PDL1 > 50%, no EGFR and ALK aberrant, received upfront pembrolizumab monotherapy, and had baseline and subsequent CT scans to assess the responses), which enabled us to study this important question. Our study had several limitations. First, it was a retrospective study based at a single cancer center with a relatively small sample size, and most of the patients included were Caucasians. Whether these findings are applicable to other patient populations remains unknown. Secondly, except for EGFR and ALK, the mutation status of many targetable mutations that may impact benefit from ICI are unknown. There is evidence indicating ROS1, RET fusion, and MET exon 14 mutations could associate with an inadequate response to ICIs despite high PD-L1 expression [32], and several specific gene alterations could also affect the efficacy of ICIs in NSCLC (e.g., KEAP1, STK11, and KRAS) [33]. As PD-L1 was only quantified as greater or less than 50% rather than the exact percentage values, we were not able to evaluate whether the PD-L1 expression has a dose–response effect on the treatment response, which has been reported [34]. A future multiple center study with a large, multiethnic patient population and more comprehensive oncopanel, a detailed PD-L1 expression is warranted to prospectively validate these intriguing findings, ensure the generalization of this observation, and provide a more robust conclusion.

## 5. Conclusions

In advanced NSCLC patients with PDL1 ≥ 50% and no EGFR and ALK aberrant, those with a no to light (≤40 pack years) smoking history and more organs involved with metastatic disease on the pretreatment CT are more likely to progress when receiving the current standard first-line treatment—pembrolizumab monotherapy. The multivariate prediction model integrating the baseline CT and clinical variables developed in this study can provide a personalized and practical tool to identify which PD-L1 high expressors have a higher likelihood of progression on pembrolizumab before commencing treatment. For this “high-risk of progression” subgroup, physicians may consider offering an alternative therapy to achieve a more effective early disease control. The model, together with the Youden cut-off, can potentially facilitate decision-making for the optimal first-line treatment in a high PD-L1 cohort.

## Figures and Tables

**Table 1:** Descriptive analysis and comparison of the baseline characteristics between two response groups—progressive disease (PD) vs. disease control (DC) after receiving pembrolizumab.

Baseline Variables	Overall, *n* = 138		Progressive Disease (PD), *n* = 46		Disease Control (DC), *n* = 92		*p*-Value
**Age [median, Q1–Q3]**							
	**73**	67–78	72	65–77	73	69–78	0.2129
**Sex** (*n*, col%)							
Female	73	52.90	21	45.65	52	56.52	0.2785
Male	65	47.10	25	54.35	40	43.48	
**Smoking status** (*n*, col%)							
Light and non-smoker	7	5.07	5	10.87	2	2.17	0.0345 *
Smoker (current/ex)	127	92.03	38	82.61	89	96.74	
Unknown	4	2.90	3	6.52	1	1.09	
**Pack years** ^1^ [median, Q1–Q3]							
	40	28–50	30	22–43	45	30–50	0.0226 *
**Smoking and pack years** (*n*, col%)							
Light and non-smoker	7	5.07	5	10.87	2	2.17	0.0031 *
Smoker with pack years ≤40	65	47.10	26	56.52	39	42.39	
Smokers with pack years >40	57	41.30	11	23.91	46	50.00	
Unknown ^2^	9	6.52	4	8.70	5	5.43	
**Eastern Cooperative Oncology Group performance status (ECOG)** (*n*, col%)							
≤1	81	58.70	20	43.48	61	66.30	0.0153 *
≥2	53	38.41	24	52.17	29	31.52	
Unknown	4	2.90	2	4.35	2	2.17	
**Tumor histology** (*n*, col%)							
Non-SCC	116	84.06	36	78.26	80	86.96	0.1923
SCC	19	13.77	9	19.57	10	10.87	
Not Otherwise Specified	3	2.17	1	2.17	2	2.17	
**On baseline CT: Number of patients with metastatic involvement in organs** (*n*, col%)							
Lung	72	52.17	27	58.70	45	48.91	0.3662
Lymph node	114	82.61	42	91.30	72	78.26	0.0612
Adrenal	33	23.91	16	34.78	17	18.48	0.0554
Liver	16	11.59	10	21.74	6	6.52	0.0119 *
Bone	48	34.78	26	56.52	22	23.91	0.0003 *
Brain ^3^	21	15.22	7	15.22	14	15.22	1.0000
Pleura ^4^	43	31.16	18	39.13	25	27.17	0.1775
**On baseline CT: Target lung lesion size in mm**^5^ [median, Q1–Q3]							
	35.5	24–50	38	24.5–51.5	35	24–47	0.4821
**On baseline CT: Number of involved organs with metastasis per patient** [median, Q1–Q3]							
	3	2–4	3	2–4	2	2–3	0.0015 *

**^1^** Restricted to the 122 smokers with known pack years. ^2^ The 9 patients with unknown data in “smoking and pack years” include 4 patients with an unknown in smoking status and 5 smokers with unknown pack years. ^3^ There were 11 patients with an unknown status of metastasis(es) present in the brain, as no brain imaging was available at the baseline. ^4^ There was 1 patient with an unknown status of metastasis(es) present in the pleura. ^5^ Restricted to 100 patients with a known target lung lesion size. * *p*-values calculated for “unknown” cases were excluded.

**Table 2:** Multivariate logistical regression analysis testing the associations between the baseline characteristics and progressive disease (i.e., PD) at FU CT while on upfront pembrolizumab.

	Univariate (Unadjusted)				Multivariate (Adjusted) ^1^			
	Odds Ratio	[95% CI for OR]		*p*-Value	Odds Ratio	[95% CI for OR]		*p*-Value
**Sex**								
Female [ref]	1.00				1.00			
Male	1.74	[0.82	3.73]	0.1495	1.92	[0.81	4.64]	0.1415
**Smoking history (smoking status and pack years)**								
0 (light and non-smoker) [ref]	1.00				1.00			
Current/ex-smoker, ≤40 pack years	0.26	[0.03	1.29]	0.1199	0.12	[0.01	0.82]	0.0412
Current/ex-smoker, >40 pack years	0.10	[0.01	0.51]	0.0092	0.04	[0.0040	0.30]	0.0033
**Eastern Cooperative Oncology Group performance status (ECOG)**								
≤1 [ref]	1.00				1.00			
≥2	2.44	[1.14	5.28]	0.0216	1.95	[0.81	4.76]	0.1357
**Age (10 years)**								
	0.70	[0.39	1.20]	0.2000	0.55	[0.28	1.07]	0.0853
**No. of involved organs on baseline CT**								
	1.67	[1.27	2.27]	0.0004	1.59	[1.19	2.20]	0.0027

^1^ To limit the number of variables in the multivariate (MV, adjusted) model, only the variables that showed a trend for a significant association (*p* ≤ 0.2) with the outcome (i.e., PD) in the univariate analysis were included. The combination of variables in the final MV model was determined using the AUC criterion, such that the selected combination achieved the best AUC across different combinations. The final MV model included age, sex, smoking pack years, ECOG, and “number of involved organs” on the baseline CT.

**Table 3:** Comparison between patients’ true responses (i.e., PD vs. DC) and their predicted “risk of progression” using the multivariate model and two cut-off values (Youden and topleft).

		Youden		Topleft	
**cut-offs on ROC to defined “high-” vs. “low-risk” of progression group**		**0.20**		0.33	
Comparison between “true response” and “predicted risk of progression”					
	True Response (PD vs. DC)	**PD (*n* = 41)**	**DC (*n* = 87)**	**PD (*n* = 41)**	**DC (*n* = 87)**
Predicted risk of progression ^1^					
High-risk of progression ^2^		38	40	29	23
Low-risk of progression ^3^		3	47	12	64
		Sensitivity:0.93	Specificity: 0.54	Sensitivity: 0.71	Specificity: 0.74

^1^ A patient’s predicted risk of progression value was calculated using Equation (1). ^2^ High-risk: if the predicted risk value is greater than the Youden or topleft cut-off value. ^3^ Low-risk: if the predicted risk value is lower than the Youden or topleft cut-off value.

## Data Availability

The data generated or analyzed during the study are available from the corresponding author by request.
